# TLR4 signalling: the key to controlling EV71 replication and inflammatory response

**DOI:** 10.3389/fcimb.2024.1393680

**Published:** 2024-06-13

**Authors:** Jinfang Hao, Hui Wang, Xiufeng Lu, Zimo Li, Xiaoyan Zhang

**Affiliations:** Department of Laboratory Medicine of Fenyang College, School of Pharmaceutical Science, Shanxi Medical University, Taiyuan, China

**Keywords:** enterovirus 71, toll-like receptor 4, inflammatory response, viral replication, immune escape

## Abstract

Hand, foot, and mouth disease (HFMD) is a common infectious disease caused by enterovirus 71 (EV71) that frequently affects children, leading to severe infections in some cases. In general, when infection occurs, the body upregulates inflammatory responses to eliminate pathogenic microorganisms to protect the host from infection. However, EV71 may inhibit host’s innate immunity to promote virus infection. At present, it is not fully understood how EV71 hijack the host cells for its own replication. Toll-like receptor 4 (TLR4), a natural immune receptor, historically associated with bacterial endotoxin-induced inflammatory responses. However, it is still unclear whether and how TLR4 is altered during EV71 infection. In this study, we observed a reduction in both TLR4 protein and gene transcript levels in RD, GES-1, and Vero cells following EV71 infection, as detected by RT-qPCR, immunofluorescence staining and western blot. Furthermore, we observed that the TLR4 downstream molecules of MYD88, p-NF-κB p65, p-TBK1 and related inflammatory cytokines were also reduced, suggesting that antiviral innate immune and inflammatory response were suppressed. To determine the impact of TLR4 changes on EV71 infection, we interfered EV71-infected RD cells with TLR4 agonist or inhibitor and the results showed that activation of TLR4 inhibited EV71 replication, while inhibition of TLR4 promote EV71 replication. Besides, EV71 replication was also promoted in TLR4 siRNA-transfected and EV71-infected RD cells. This suggests that down-regulation the expression of TLR4 by EV71 can inhibit host immune defense to promote EV71 self-replication. This novel mechanism may be a strategy for EV71 to evade host immunity.

## 1 Introduction

Hand-foot-and-mouth disease (HFMD) is a common childhood infectious disease, primarily caused by Enterovirus 71 (EV71). In severe cases, HFMD can result in serious neurological complications such as brainstem encephalitis, neurogenic pulmonary oedema, and even death (Lu et al., 2021). EV71 is a single-stranded positive-sense RNA virus, and belongs to the small RNA virus family, genus Enterovirus. Its genome is approximately 7.4 kb in size and encodes four viral capsid structural proteins (VP1-VP4) along with seven non-structural proteins (2A-2C and 3A-3D). The expression level of the VP1 protein can reflect the replication of EV71 (Li et al., 2018; Liu et al., 2019).

Inflammation, a natural response of the immune system, plays a crucial role in fighting pathogen invasion and serves as the first line of defense against pathogen infection (Riaz et al., 2023). It guides immune cells to the site of infection through vasodilation, increased vascular permeability, and the release of inflammatory mediators. Subsequently, immune cells engulf pathogens, release antimicrobial substances, and mobilize other cells to participate in combating the infection (van der Meijden and Heemskerk, 2018). However, certain pathogens have evolved strategies to inhibit the production of inflammatory mediators, leading to chronic infection or evasion of the host immune system. Research has shown that Mycobacterium tuberculosis and Salmonella are able to evade the immune system by suppressing the production of inflammation cytokines such as interferon-gamma (IFN-γ), interleukin-1beta (IL-1β), and tumor necrosis factor-alpha (TNF-α) (Kogut et al., 2020; Ravesloot-Chávez et al., 2021; Zhou et al., 2021; Kaur et al., 2022; Shariq et al., 2022). Similarly, studies have demonstrated that human immunodeficiency virus, hepatitis B virus, and influenza A virus can evade the host immune response by inhibiting the production of inflammation cytokines (Bergantz et al., 2019; Megahed et al., 2020; Zhao et al., 2022). The innate immune response, particularly type I interferons (IFN-I), plays a crucial role in limiting the EV71 replication (Coyne et al., 2017). Clinical studies have shown that the expression of IFN-I is suppressed in the bodies of children infected with EV71 (Zou et al., 2015). Lei Xiaobo, Wang Bei et al. conducted studies on the relevant mechanism and found that EV71 non-structural protein 3C prevented the antiviral and immunomodulatory activity of the interferon response (Lei et al., 2010), and further studies reported that the EV71 3C protein inhibited the TLR3-mediated interferon response by down-regulating TRIF (Lei et al., 2011). Additionally, the EV71 non-structural protein 2Apro blocks the IFN-I pathway to inhibit the inflammatory response and facilitate viral replication (Coyne et al., 2013). Researchers also found that EV71 3C interacted with TAB2 and TAK1 to inhibit the activation of the NF-κB pathway. The EV71-mediated cleavage of TAK1/TAB1/TAB2/TAB3 may be one of the mechanisms inhibiting the inflammatory response (Lei et al., 2014). Clinical studies have reported a strong correlation between TLR4 gene polymorphism and severe enterovirus 71 infection (Chen et al., 2020). We speculate that TLR4 plays an important role in EV71 infection and this study aims to elucidate this role.

Toll-like receptors (TLRs) are key pattern recognition receptors that play a vital role in innate immunity (Duan et al., 2022) and also participate in adaptive immune responses (Xing et al., 2017; Kaur et al., 2022). Among the Toll-like receptor family, TLR4 is a transmembrane protein that is distributed in various tissue cells such as mononuclear macrophages, epithelial cells, and dendritic cells. Studies have shown that the TLR4 receptor plays an important role in the regulation of the body’s inflammatory response (Hong et al., 2020; Rong et al., 2021). Specifically, the TLR4 receptor is involved in most Gram-negative bacterial lipopolysaccharide (LPS)-induced signal transduction, where LPS activation of TLR4 triggers the production of intracellular inflammatory cytokines, thereby activating the innate immune system (Ciesielska et al., 2020; Mazgaeen and Gurung, 2020; Olona et al., 2021). TLR4 activates downstream inflammatory pathways in a MYD88-dependent or MYD88-independent manner (Rong et al., 2021). MyD88 serves as a central adapter protein that recruits and phosphorylates downstream proteins to activate the NF-kB and MAPK pathways, leading to increased expression of interleukin-6 (IL-6), macrophage inflammatory protein, TNFα. Elevated levels of TNF-α and IL-6 can further activate TLR4 to regulate the NF-kB and STAT3 signaling pathways (Wu et al., 2022a).

Researchers have explored the role of TLR4 in the innate immune response to viruses, but the mutual regulatory effects between viruses and TLR4 remain uncertain. Moreover, research has indicated that the TAT protein of HIV-1 can also be recognized by TLR4 on human dendritic cells and monocyte/macrophages, activating the NF-κB pathway and inducing the production of inflammatory cytokines (Ben Haij et al., 2015). TLR4 signaling can also promote the production of IL-10, which facilitates the replication of Mammary Tumor Virus(MMTV)in mice, thereby allowing the virus to persist (Kane et al., 2011). The changes in TLR4 can also be observed during infection with respiratory syncytial virus (RSV), dengue virus (DENV), and Ebola virus (EBOV) infection (Kuhn et al., 2019; Voelker and Numata, 2019; Halajian et al., 2022). However, research has also shown that the TLR4 signaling can protect mice from vaccinia virus infection by limiting viral replication and local inflammation (Buller et al., 2008). The TLR4 agonist LPS can significantly inhibit lung inflammation, mucus production, airway inflammatory cell infiltration caused by FI-RSV infection (Yin et al., 2019). Interestingly, recent clinical studies have revealed that the average expression level of TLR4 mRNA in peripheral blood monocytes of children with severe hand, foot, and mouth disease caused by EV71 is significantly higher than in other children (Zhu et al., 2017). Furthermore, EV71 virus-like particles induce the activation and maturation of human monocyte-derived dendritic cells through TLR4 signaling transduction (Stoddart et al., 2014). Both clinical studies (Zou et al., 2015) and cellular level investigations by Lei Xiaobo (Lei et al., 2010) and Wang Bei (Coyne et al., 2013) have shown that EV71 causes the downregulation of IFN-I. Given that IFN-I is known to be downstream of TLR4, the changes in TLR4 during EV71 infection need to be further investigated. Further study of the interaction between EV71 and TLR4 will help to elucidate the regulatory mechanism of the virus’ immune response to the host and provide an important theoretical basis for the development of novel antiviral strategies.

## 2 Materials and methods

### 2.1 Cell culture

Human rhabdomyosarcoma cells (RD), normal human gastric epithelial cells (GES-1) and African green monkey kidney cells (Vero) were preserved at the Fenyang College of Shanxi Medical University (Fenyang, China). EV71 (Fuyang-0805) was provided by Prof. Zhendong Zhao, Institute of Pathogenic Biology, Chinese Academy of Medical Sciences and Peking Union Medical College. Cells were inoculated into Minimum Essential Medium (MEM) and Dulbecco’s Modified Eagle Medium (DMEM) supplemented with 10% fetal bovine serum and 1% penicillin and streptomycin, respectively. The cells were then cultured in a 5% CO_2_ incubator at 37°C. RD cells were infected with EV71 for 0.5 h, 1 h, 1.5 h, and 2 h and then treated with 1µg/mL LPS (TLR4 agonist, S1732, Beyotime) or pretreated with 10 nM TAK-242 (TLR4 inhibitor, HY-11109, MCE) for 15 min and then infected with EV71 for 6 h.

### 2.2 Observation of cell morphology

RD cells were inoculated into 6-well culture plates (1×10^6^ cells/well) and infected with EV71 at different MOIs (0, 1, 3 or 5) for 6 h. The morphological changes of the RD cells were observed under an inverted microscope (Nikon, Japan).

### 2.3 Western blot

RD, GES-1 and Vero cells were infected with EV71 for 2 h, 4 h, or 6 h and the cells were collected in a centrifuge tube at 2000 ×g for 3 minutes. Total protein was extracted using RIPA lysis buffer containing protease inhibitor and phosphatase inhibitor (Beyotime). The protein concentration of the sample was determined using the BCA protein detection kit (Boster). Subsequently, SDS-PAGE protein loading buffer (1×) was added, and the protein was denatured by heating at 95°C for 10 min. After separation by 10% SDS-PAGE gel, the proteins were transferred to nitrocellulose (NC) membrane. The membrane was then incubated with 5% skim milk powder at room temperature for 2 h, followed by incubation with specific antibodies including TLR4 (1:1000, WL00196, Wanleibio), NLRP3 (1:2000, WL02635, Wanleibio), COX2 (1:2000, WL01750, Wanleibio), IL-6 (1: 1000, WL02841, Wanleibio), IFN-γ (1:1000, WL02440, Wanleibio), β-actin (1:5000, BM0005, ABclonal), VP1 (1:1000, PAB7631-D01P, Abnova), TBK1 (1:1000, BM4038, Boster), p-TBK1 (1:1000, P00261, Boster) overnight at 4°C. After incubation, the primary antibody was removed, and the membrane was washed with phosphate buffer (PBS) before being incubated with goat anti-rabbit IgG (1:8000, CW0103, Boster) for 2 hours at room temperature. Following another wash with PBS, a highly sensitive ECL (AR1171, Beyotime) was added for visualization of the target proteins.

### 2.4 Real-time quantitative PCR

Total RNA was extracted from RD, GES-1 and Vero cells using TRIzol (Mei5). Subsequently, cDNA was synthesized from 1µg of RNA using the BeyoRTTM II cDNA first strand synthesis kit (D7168M, Beyotime). Real-time quantitative PCR (RT-qPCR) was then performed using Hieff qPCR SYBR Green Master Mix (Yeasen, Shanghai, China). The PCR reaction mixture contained of 10μL 1×SYBR Green Mix, 0.4μL specific primer, 1μL cDNA and DEPC water, with a total volume of 20μL. The amplification of target fragments involved an initial denaturation at 95°C for 2 minutes, followed by denaturation at 95°C for 10 s, annealing at 55°C for 30 s, and 40 cycles. TLR4 mRNA levels were analyzed by 2^-ΔΔCt^. The primers were used for this study as follows: *TLR4* forward, 5′-TCTTGGTGGAAGTTGAACGAATGG-3′ and reverse, 5′- AGCACACTGACCGACAC-3′; *EV71* forward, 5′- CGCACAGGGTCACTCAGAAC-3′ and reverse, 5′- GCCCATTGCCACCAGTAGAC-3′; *GAPDH* forward, 5′- ACAACTTTGGCATTGTGGAA-3′ and reverse, 5′- GATGCAGGGATGATGTTCTG-3′.

### 2.5 Immunofluorescence staining

After EV71 infection of RD, Vero and GES-1 cells for 6 hours, the cells were washed with PBS and then fixed with 4% paraformaldehyde at 4°C for 20 min. Subsequently, after another wash with PBS, the cells were treated with 5% bovine serum albumin (BSA) at room temperature for 1 hour, followed by incubation with the TLR4 antibody at 4°C overnight. Cy3-labeled goat anti-rabbit IgG antibody (A0423, Beyotime) was then incubated for 1 hour at room temperature. The nucleus was stained with 4’, 6-diaminidine-2-phenylindole (DAPI, C1005, Beyotime) for 5 minutes at room temperature. Finally, the images were photographed using a fluorescence microscope (Nikon, Japan).

### 2.6 TCID_50_

RD cells were seeded into 96-well plates at a density of 1.0×10^4^ cells/well. The following day, the virus was serially diluted 10-fold and inoculated into the 96-well plate. Each dilution was added to a series of 8 wells, with each well containing 100 µL. At the same time, normal cells were used as controls. Cells were cultured at 37°C in a 5% CO_2_ incubator (Thermo Fisher, Germany) and observed under a microscope for 5 to 7 days. The observation results were recorded and statistical analysis was performed.

### 2.7 Cell transfection

The non-target siRNA and TLR4 siRNA were constructed by Genepharma Co., Ltd. (Suzhou, China) and then transfected into RD cells using Lipofectamine 8000 (C0533- 0.5 mL; Beyotime, Shanghai, China) following to the manufacturer’s protocol.

### 2.8 Statistical analysis

All experimental data were independently repeated at least three times, and statistical analysis was performed using GraphPad Prism 8.0.2 software (CA, USA). One-way ANOVA was used to compare multiple groups, and T-test was used to compare statistical differences between two groups. The Data is presented as mean ± standard (SD) deviation. *P* < 0.05 was considered statistically significant.

## 3 Result

### 3.1 EV71 infection causes cytopathies

**Figure 1:**
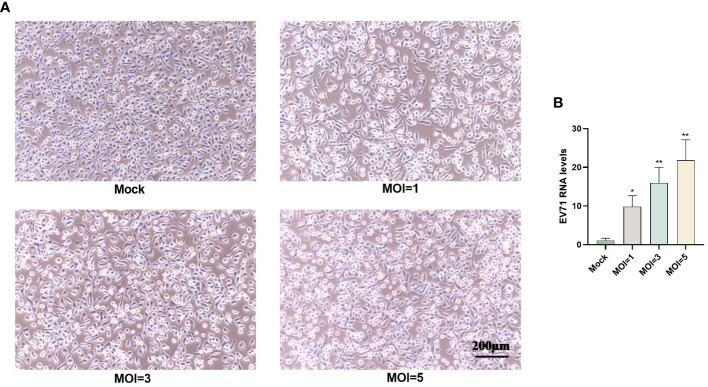
EV71 infection causes cytopathies. **(A)** RD cells were infected with EV71 at different MOIs for 6 hours and observed using an inverted microscope. **(B)** RT-qPCR analysis was performed to measure EV71 RNA levels in EV71-infected RD cells. Data are presented as the mean ± SD. *P < 0.05 and **P < 0.01 vs control group.

The RD cells were infected with EV71 at different MOIs (0, 1, 3 or 5) for 6 h and pathological changes were observed using inverted microscopy. The results indicated that RD cells in the EV71 group exhibited shrinkage and an increased number of floating cells compared to the mock group (**Figure 1A**). Additionally, EV71 RNA levels were measured in RD cells infected with EV71 (MOI=0, 1, 3 or 5), revealing a dose-dependent increase in EV71 RNA levels (**Figure 1B**).

### 3.2 EV71 infection caused the downregulation of TLR4

**Figure 2:**
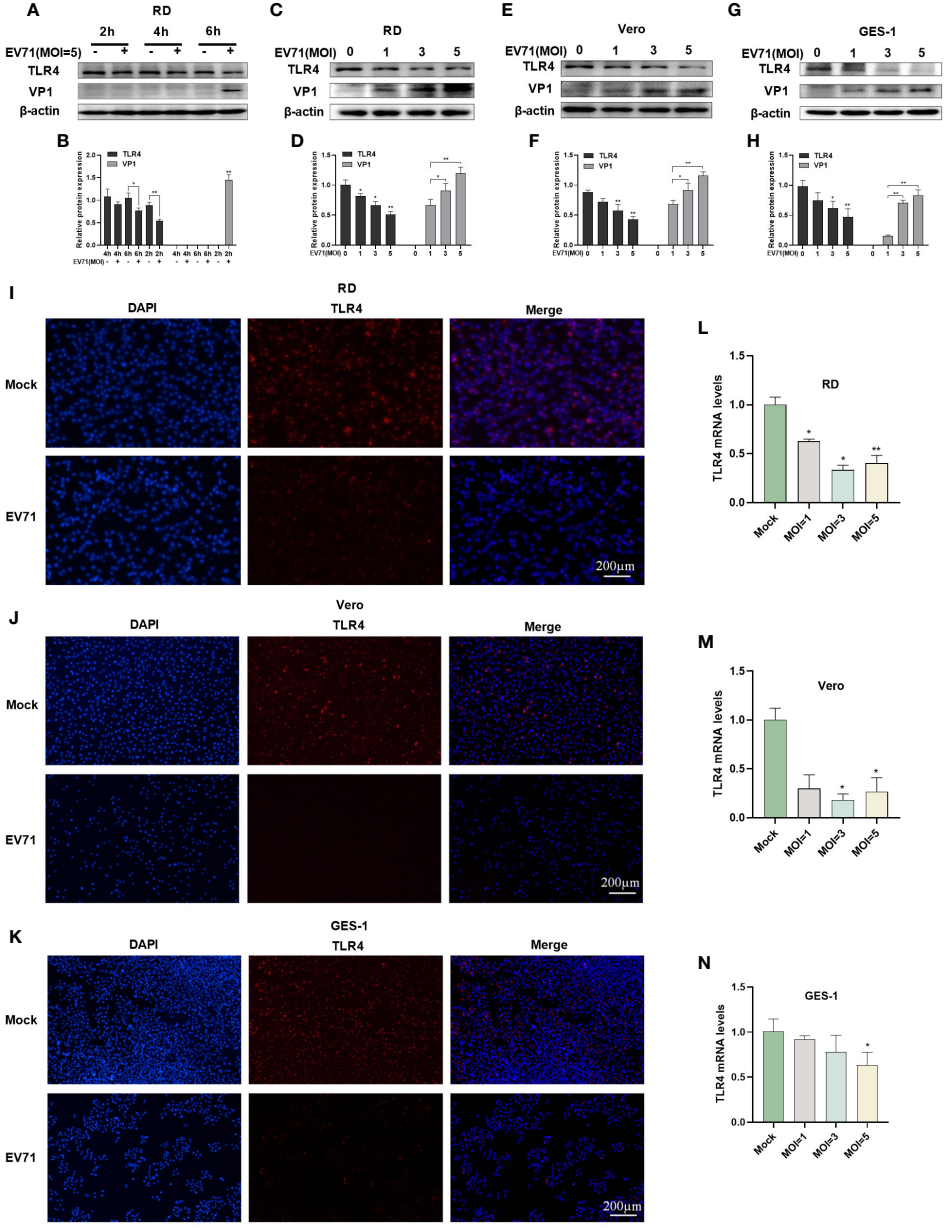
EV71 infection caused the downregulation of TLR4. **(A, B)** Western blot analysis was performed to detect the changes in TLR4 and VP1 levels in EV71-infected RD cells at various time points; Western blot analysis was performed to detect the protein levels of TLR4 and VP1 in EV71-infected **(C, D)** RD cells, **(E, F)** Vero cells and **(G, H)** GES-1 cells at different MOIs; RT-qPCR analysis was used to detect TLR4 mRNA levels in EV71-infected **(L)** RD, **(M)** Vero and **(N)** GES-1 cells; Immunofluorescence analysis showing the TLR4 protein expression in EV71-infected **(I)** RD, **(J)** Vero and **(K)** GES-1 cells. Data are presented as the mean ± SD. *P < 0.05 and **P < 0.01 vs control group.

RD cells were infected with EV71 at 2, 4, and 6 h and the protein expression of TLR4 and VP1 was measured by western blot. The results revealed that the EV71 structural protein VP1 was detected at 6 hours, indicating this time point as a critical early stage for EV71 infection. Interestingly, a decrease in TLR4 protein levels was observed at 4 hours, with a significant reduction at 6 hours (**Figures 2A, B**).

Subsequently, TLR4 protein levels were investigated at 6 hours post EV71 infection in different cell models. Western blot analysis in RD cells (**Figures 2C, D**), Vero cells (**Figures 2E, F**), and GES-1 cells (**Figures 2G, H**) revealed a progressive decrease in TLR4 protein expression with a gradual increase in the EV71 structural protein VP1. TLR4 mRNA levels were also measured via RT-qPCR and the results showed a significant decrease in TLR4 mRNA levels in EV71-infected RD (**Figure 2L**), Vero (**Figure 2M**), and GES-1 (**Figure 2N**) cells at 6 hours. Immunofluorescence results further confirmed the reduction of TLR4 expression in RD (**Figure 2I**), Vero (**Figure 2J**) and GES-1 (**Figure 2K**) cells at 6 hours after EV71 infection. In conclusion, EV71 can suppress TLR4 gene transcription and protein expression in host cells.

### 3.3 EV71 infection inhibits the TLR4/MYD88/NF-κB and TBK1 pathway

**Figure 3:**
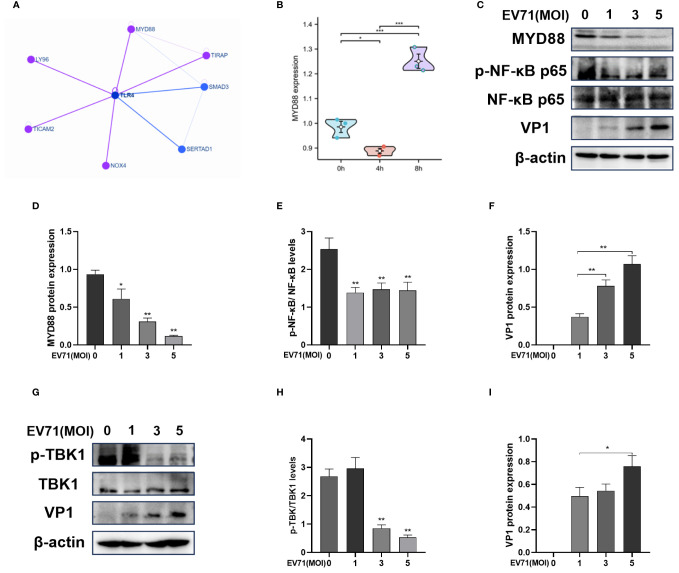
EV71 infection inhibits the TLR4/MYD88/NF-κB and TBK1 pathway. **(A)** The network of TLR4 interacting proteins; **(B)** MYD88 gene expression of the GSE15323 dataset from the GEO database; **(C–F)** Western blot analysis of MYD88, p-NF-κB p65, NF-κB p65 and VP1 protein expression in EV71 infected RD cells at different MOIs. **(G–I)** Western blot analysis of p-TBK1/TBK1 and VP1 protein expression in EV71 infected RD cells at different MOIs. Data are presented as the mean ± SD. *P < 0.05, **P < 0.01, ***P < 0.001 vs control group.

MYD88 is an important adaptor in the downstream of TLR4. We identified possible interacting proteins of TLR4 via the HINT database (**Figure 3A**), in which TLR4, MYD88 and TIRAP regulate the NF-κB signaling pathway. By analyzing the GSE15323 dataset from the GEO database, which contains 9 samples of human RD cells under different conditions (uninfected, EV71 infection for 4 h, and EV71 infection for 8 h) and we observed a reduction in MYD88 gene expression in RD cells infected with EV71 for 4 h (**Figure 3B**). Therefore, we suggest that EV71 may regulate the NF-κB signaling pathway through MYD88. We then carried out experimental verification and infected RD cells with EV71 at different MOIs for 6 h (**Figures 3C, F**). As the viral load increased, the protein expression levels of MYD88 were gradually decreased (**Figures 3C, D**). The p-NF-κB p65/NF-κB p65 levels were also decreased following EV71 infection for 6 h, indicating that EV71 infection inhibited NF-κB activation (**Figures 3C, E**). In addition, we measured the protein level of p-TBK1/TBK1 and the results showed that p-TBK1/TBK1 level was decreased in EV71-infected RD cells for 6 h (**Figures 3G–I**), indicating that EV71 infection inhibited the innate immune response. In conclusion, our results suggest that EV71 infection suppresses the TLR4/MYD88/NF-κB and TBK1 signaling pathway.

### 3.4 EV71 infection inhibited inflammatory response

**Figure 4:**
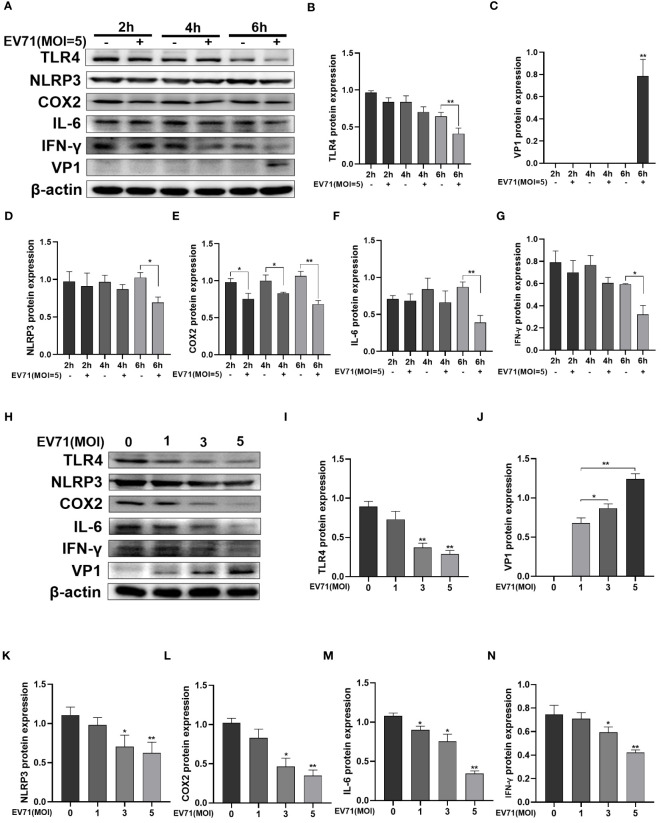
EV71 infection inhibited inflammatory response. **(A–G)** Western blot analysis was performed to detect the changes of inflammation-related proteins in EV71-infected RD cells at different time points; **(H–N)** Western blot analysis of the changes of inflammation-related proteins in EV71-infected RD cells at different MOIs. *P < 0.05 and **P < 0.01 vs control group.

We hypothesize that in the initial 6 hours of EV71 infection, the downregulation of TLR4 expression suppresses the inflammatory response and helps the virus to evade host immunity. Western blot analysis was performed following EV71 infection of RD cells for 2 h, 4 h, and 6 h (**Figure 4A**). VP1 expression was detectable starting from 6 h (**Figure 4C**), while TLR4 expression was significantly decreased after EV71 infection at 6 h (**Figure 4B**). The inflammatory mediators NLRP3, COX2, IL-6, and IFN-γ were all downregulated after 6 h of EV71 infection (**Figures 4D–G**).

Next, RD cells were infected with EV71 for 6 hours at MOIs of 1, 3, and 5 (**Figure 4H**). With increasing MOIs, VP1 expression showed a gradual increase (**Figure 4J**), while TLR4 expression showed a gradual decrease (**Figure 4I**). Levels of inflammatory cytokines NLRP3, COX2, IL-6 and IFN-γ were also decreased with increasing MOIs (**Figures 4K–N**). These results indicate that EV71 infection for 6 h effectively suppressed the inflammatory response.

### 3.5 Activated TLR4 recovered the inflammation and inhibited EV71 replication

**Figure 5:**
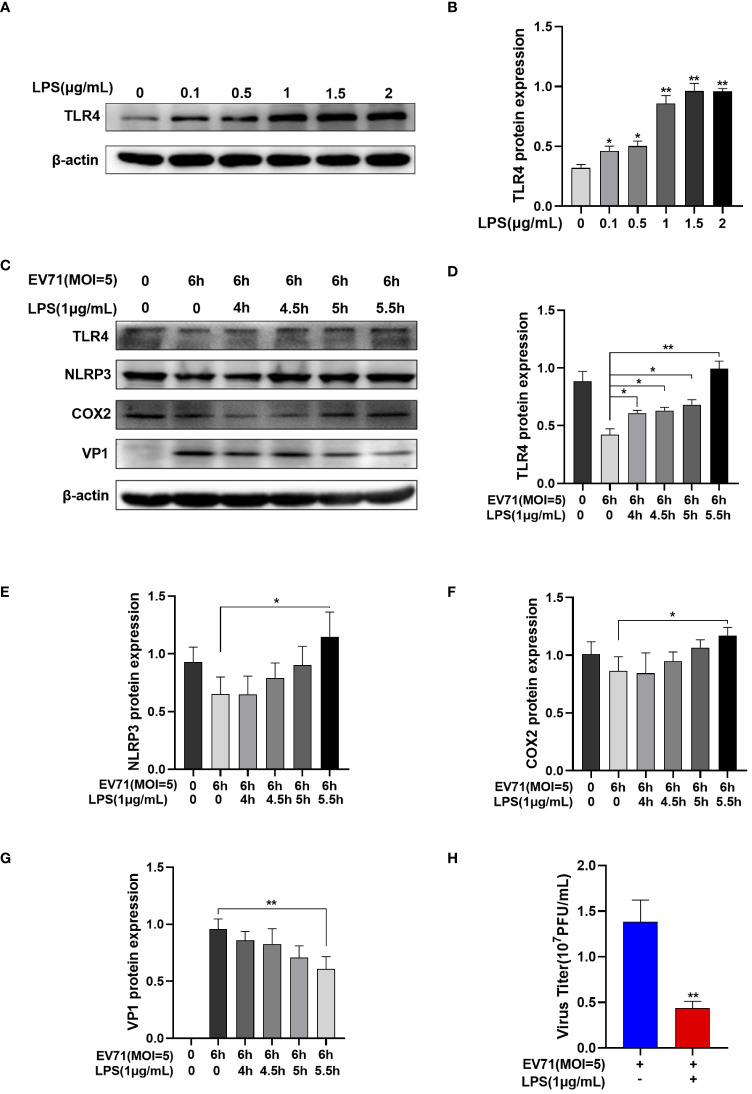
Activated TLR4 recovered the inflammation and inhibited EV71 replication. **(A, B)** Western blot analysis was performed to detect the changes of TLR4 protein after different doses of LPS intervention; **(C–G)** Western blot analysis of the protein expression of TLR4, NLRP3, COX2 and VP1 in EV71-infected and LPS-treated RD cells; **(H)** TCID_50_ detects the virus titer of the supernatant in different groups. Data are presented as the mean ± SD. *P < 0.05 and **P < 0.01 control group.

EV71 infection leads to the downregulation of TLR4. We aim to further investigate whether TLR4 activation affects EV71 replication. Lipopolysaccharide (LPS) is commonly used as a TLR4 agonist. Following treatment with different doses of LPS for 4 hours, TLR4 protein levels gradually increase in a dose-dependent manner in RD cells (**Figures 5A, B**).

RD cells were infected with EV71 for 0.5 h, 1 h, 1.5 h, and 2 h and then LPS was added. RD cells were infected with EV71 for a total of 6 h and harvested. Western blot analysis revealed that EV71 infection of RD cells led to the downregulation of TLR4 (**Figure 5C**). However, with extended exposure to LPS, the EV71’s inhibitory of TLR4 was reversed (**Figure 5D**), resulting in an increase in inflammation cytokines (**Figures 5E, F**) and a decrease in the EV71 structural protein VP1 (**Figure 5G**). At the same time, the viral titers of the supernatant were decreased in the LPS and EV71 group compared to the EV71 group, indicating a reduction in viral release (**Figure 5H**). In conclusion, TLR4 activation inhibits EV71 replication.

### 3.6 The inhibition of TLR4 promotes EV71 replication

**Figure 6:**
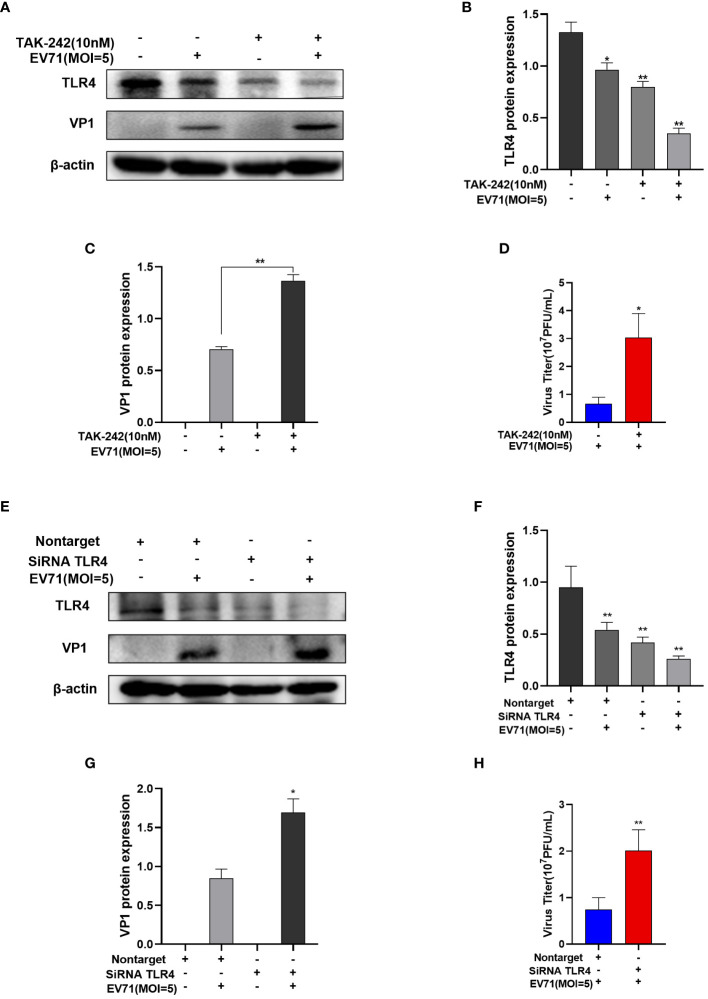
The inhibition of TLR4 promotes EV71 replication. **(A–C)** Western blot analysis of TLR4 and VP1 protein levels in EV71-infected and TAK-242-treated RD cells; **(D)** TCID_50_ detects the virus titer of the supernatant in the EV71-infected group and the EV71-infected and TAK-242-treated group. **(E–G)** Western blot analysis of TLR4 and VP1 protein levels in TLR4 siRNA transfected and EV71 infected RD cells; **(H)** TCID_50_ detects the virus titer of the supernatant in TLR4 siRNA transfected and EV71 infected RD cells. Data are presented as the mean ± SD. *P < 0.05 and **P < 0.01 vs control group.

TAK-242 is a TLR4 inhibitor (Ii et al., 2006). RD cells were pre-treated with TAK-242 for 15 min prior to EV71 infection for 6 h. Western blot results showed that TLR4 protein levels were decreased in the TAK-242 group compared with control group with or without EV71 infection (**Figures 6A, B**), leading to an increase in VP1 protein levels (**Figure 6C**). Concurrently, viral titer of the supernatant was increased after TAK-242 treatment of EV71-infected RD cells (**Figure 6D**). Besides, RD cells were transfected with non-target siRNA or TLR4 siRNA for 72 h and then infected with EV71 for 6h (**Figure 6E**). The VP1 protein level (**Figure 6G**) and the virus titer (**Figure 6H**) of the supernatant were increased in TLR4 siRNA transfected and EV71 infected RD cells compared with non-target siRNA transfected and EV71 infected RD cells. It suggests that down-regulates TLR4 expression to promote EV71 replication (**Figure 6F**). In conclusion, the inhibition of TLR4 facilitates EV71 replication.

## 4 Discussion

TLR4, as one of the pattern recognition receptors, plays a crucial role in activating of the NF-kB inflammatory pathway (Wu et al., 2022b) and antiviral immune response (Bierwagen et al., 2023). While traditionally associated with bacterial LPS recognition, recent research highlights its significance in viral infections. For instance, during Chikungunya virus infection, the interaction between TLR4 and the envelope protein E2 acts as a vital entry point for the virus into the host (Mahish et al., 2023). Our research has revealed that EV71 infection leads to the downregulation of TLR4. Specifically, 6 hours for EV71 infection, the EV71 structural protein VP1 was detected and showed in a dose-dependent increase. Moreover, a reduction in TLR4 protein levels was noted in EV71-infected RD, GES-1, and Vero cells for 6 h. This indicates that the pattern recognition receptor TLR4 can be regulated by EV71. EV71 infection can interfere the host cell’s immune response through various mechanisms to promote virus replication and spread. Studies have indicated that children infected with EV71 may experience suppression of innate immune, possibly due to interferon blockade (Coyne et al., 2013; Lei et al., 2013). Mice lacking interferon receptors and infected with EV71 exhibit symptoms such as limb paralysis and mortality, indicating the crucial role of interferons in preventing EV71 infection and disease progression (Liou et al., 2016; Shih et al., 2018). Furthermore, the immune-related protein TRAF3 interacting protein 3 (TRAF3IP3) has been shown to inhibit EV71 replication, while the non-structural protein 3C of EV71 can counteract this inhibition through protein cleavage (Li et al., 2022). We speculate that the EV71-induced reduction of TLR4 may serve as an immune evasion strategy to promote its own replication.

Continuing our investigation into the downstream pathway of TLR4, we found that EV71 infection leads to the downregulation of TLR4 while concomitantly inhibiting the TLR4/MYD88/NF-κB and TBK1 pathway. MYD88, a crucial adaptor protein in TLR4 signaling, plays a pivotal role in the activation of the TLR4 signaling pathway (Zhou et al., 2017; Xu et al., 2022). Our results show that EV71 infection interferes the TLR4 signaling pathway by inhibiting the MYD88 activation and downstream NF-κB phosphorylation. Furthermore, studies have shown that EV71 infection can suppress the production of various inflammatory factors, such as IL-6 (Tanaka et al., 2014), NLRP3 (Zhang et al., 2023), COX2 (Gupta et al., 2018) and IFN-γ (Nakamura-Shinya et al., 2022), thereby dampening the immune response and these findings are consistent with our results. In addition, we find that EV71 infection also inhibits the p-TBK1/TBK1 protein level to suppress the antiviral innate immune response. Our results provide further evidence that EV71 may weaken the host cell immune response by inhibiting the TLR4 expression, thereby promoting its own replication and spread.

We speculate that the inhibition of inflammation may affect viral replication. Research has shown that during the early stages of porcine reproductive and respiratory syndrome virus infection, the TLR4-NF-κB pathway was activated by LPS treatment and significantly increased the expression of inflammatory cytokines to inhibit virus replication (Zhu et al., 2020). Upon activating TLR4 with LPS, the inhibitory effects of EV71 on TLR4 and inflammation response were reversed in our study. As a result, the EV71 structural protein VP1 and virus titers of the supernatant were decreased in EV71-infected and LPS-treated RD cells. Subsequently, we treated EV71-infected RD cells with the TLR4 inhibitor TAK-242 or transfected them with the siRNA TLR4 and found that inhibition of TLR4 expression resulted in an increase in EV71 structural protein VP1 and virus titers of the supernatant, suggesting that inhibition the TLR4 expression can promote EV71 replication. This result supports our speculation. Recent studies have shown that overexpression of TLR4 and TLR2/TLR4 heterodimers in cells can activate innate responses and inhibit EV71 replication, which is consistent with our research.

We are making efforts to further explore the specific mechanism by which EV71 downregulates TLR4 expression, which sheds light on the viral infection process. Interestingly, we have found that EV71 infection not only impacts the protein levels of TLR4 but also reduces TLR4 mRNA expression. This may involve interactions between viral proteins and host cell transcription factors, affecting the transcriptional regulation of the TLR4 gene. On one hand, as a single-stranded RNA virus, EV71 can influence the transcriptional regulation of host cells. Studies have shown that EV71 down-regulated microRNA-302, leading to the up-regulate of the protein level of KPNA2, which in turn inhibits EV71-induced innate immune responses (Peng et al., 2018). Additionally, EV71 induces miR-124 to partially inhibit host STAT3 and IL-6R antiviral activity (Chang et al., 2017). NF-κB is a crucial downstream molecule of the TLR4 signaling pathway, playing a key role in inflammatory response. We have observed that EV71 infection inhibited NF-κB phosphorylation to reduce the production of inflammatory factors. Furthermore, we speculate that NF-κB, as a transcription factor, may be involved in TLR4 transcriptional regulation. On the other hand, the 2A and 3C enzymes, important proteases of the EV71 virus, can cleave various host cell signaling molecules and immune-related proteins, disrupting cell signaling pathways and inhibit cytokine and interferon production as well as interferon-stimulated gene expression. Research has shown that the EV71 non-structural protein 3C protease promotes viral protein maturation by cleaving multiple protein precursors and also impact host innate immune proteins to suppress antiviral immune responses (Li et al., 2022). EV71 virus protein 2A can cleave MAVS to inhibit the type I IFN production (Coyne et al., 2013). Additionally, the cleavage of interferon regulatory factor 7 by EV71’s 3C protein allows the virus to evade cellular immune responses (Lei et al., 2013). Therefore, we speculate that EV71 may interfere with host cell immune responses through its 2A and 3C enzymes. It is worth noting that the transcriptional regulation of TLR4 in the early stage of EV71 infection remains incompletely understood. We will uncover the interaction between EV71 and TLR4 in depth to understand EV71’s regulatory mechanisms on host immune responses in further research.

In this study, we revealed that EV71 infection downregulated the expression of TLR4 to concomitantly suppress the expression of inflammatory cytokines, indicating that this may be one of the new mechanisms of immune evasion by EV71. The regulation to TLR4 could be served as a new strategy to control EV71 infection, providing a reference for antiviral research.

## Data availability statement

The original contributions presented in the study are included in the article/supplementary material. Further inquiries can be directed to the corresponding author.

## Ethics statement

Ethical approval was not required for the studies on humans in accordance with the local legislation and institutional requirements because only commercially available established cell lines were used.

## Author contributions

JH: Data curation, Methodology, Software, Validation, Visualization, Writing – original draft. HW: Data curation, Validation, Visualization, Writing – review & editing. XL: Software, Validation, Writing – review & editing. ZL: Methodology, Writing – review & editing. XZ: Conceptualization, Funding acquisition, Project administration, Supervision, Writing – review & editing.
